# Associations Between Sprint Mechanical Properties and Change of Direction Ability and Asymmetries in COD Speed Performance in Basketball and Volleyball Players

**DOI:** 10.3390/life14111434

**Published:** 2024-11-06

**Authors:** Zhihao Zhang, Mingchen Jiang, Yifan Jing, Mingjia Li, Yanchun Li, Xiaolin Yang

**Affiliations:** 1 Sport Science School, Beijing Sport University, Beijing 100084, China; zhangzhihao@bsu.edu.cn; 2 China Volleyball College, Beijing Sport University, Beijing 100084, China; jiangmingchen@cup.edu.cn; 3 College of Physical Education and Arts Humanities, China University of Petroleum, Beijing 100100, China; 4 China Institute of Sport and Health Science, Beijing Sport University, Beijing 100084, China; 1004320180587@bsu.edu.cn (Y.J.); yangxiaolin@bsu.edu.cn (X.Y.); 5 Caofeidian College of Technology, Tangshan 063205, China; 6 China Basketball College, Beijing Sport University, Beijing 100084, China; limingjia@bsu.edu.cn; 7 Beijing Key Laboratory of Sports Performance and Skill Assessment, Beijing 100084, China; 8 Key Laboratory for Performance Training & Recovery of General Administration of Sport, Beijing 100084, China

**Keywords:** force–velocity profile, agility performance, asymmetries in COD speed performance

## Abstract

This study aimed to assess the associations between sprint force–velocity profile variables with change of direction (COD) performance and to investigate the impact of these variables on asymmetries in COD speed performance. Ninety-nine participants (volleyball players: n = 44, basketball players: n = 55) performed 40 m sprints for Fv relationship calculation, two COD tests (Modified Agility T-test and 505 test). A partial least squares (PLS) regression analysis was conducted to determine the relationships between the variables. The V_0_ was the most influential variable; it was negatively associated with COD performance variables (β = −0.260, −0.263 and −0.244 for MAT, 505-D and 505-ND, respectively), and F_0_ (β = 0.169, 0.163) was associated with the COD performance variables (COD deficit D and COD deficit ND, respectively), slightly larger than the effects of Fv_slope_ (β = −0.162, −0.146), D_RF_ (β = −0.159, −0.142) and P_max_ (β = −0.162, −0.146). For COD deficit imbalance, the D_RF_ (β = −0.070) was the most influential variable followed by Fv_slope_ (β = −0.068), F_0_ (β = 0.046) and gender (β = 0.031). V_0_ and RF_max_ were the critical variables for improving COD performance that includes linear sprints, while D_RF_, Fv_slope_, F_0_ and P_max_ collectively influence 180° COD performance. Meanwhile, D_RF_ and Fv_slope_ were important factors for asymmetries in COD speed performance. It is recommended to use the Fv profile to diagnose different COD movement patterns and then develop training plans accordingly for team sports played on smaller courts, such as basketball and volleyball.

## 1. Introduction

Team sports players must frequently perform change of direction (COD) movements such as running back and forth repeatedly. Compared to linear sprinting, COD movements involve additional acceleration and deceleration to overcome inertia and quickly generate propulsive forces in new directions. The ability to change direction is particularly critical in basketball and volleyball [1,2,3]. Characteristic gameplay in basketball involves short and rapid offenses, with an average maximum movement distance of 9.48 m [4]. In volleyball, 83.7% of game durations are less than 10 s. Regarding covered distance, 45.7% of movements range between 5 and 10 m, while 85.3% are less than 15 m [5]. In both aforementioned sports, due to the small area of the competition court and the frequent changes in competition intensity, athletes are required to repeatedly engage in high-intensity exercise accompanied by frequent COD movements [4,5].

Due to the similarity between the movement patterns of the Modified Agility Test (MAT) and the sprinting and lateral movement demands in volleyball and basketball, as well as its ability to better replicate the typical multi-directional, high-intensity movement distances in team sports, a series of studies have used the MAT to assess the COD performance of basketball and volleyball players [6,7,8,9]. The 505 COD test also widely used to measure COD performance for each leg in volleyball and basketball [10,11]. Time is recorded at the 10-m mark of the sprint and ends with the 5-m sprint following the turn. This timing omits the acceleration process from a stationary start, reflecting the phase where athletes move at higher speeds during a game [10]. However, the relevance of the total time of the 505 COD test in evaluating COD ability has recently been questioned, as literature shows that only 31% of the 505 COD test time is devoted to changing direction [12,13]. Therefore, to minimize the influence of physical fitness (body weight, size and body’s center of gravity), linear speed and acceleration on 505 COD test results, a COD deficit, which is an evaluation method for COD ability—where a participant’s 10 m time is subtracted from the 505 COD time—is considered an effective method to independently assess COD ability while avoiding these confounding factors [2,5,14,15]. The MAT and 505 COD test provide a comprehensive assessment of multidirectional (90° and 180°) movement, such as a 180° backdoor cut to sprint away from defensive pressure, with performance influenced by the linear and lateral speeds [10]. Conversely, COD deficit offers an isolated evaluation of COD performance [16].

In recent years, studies of lower limb asymmetry in sports performance have garnered considerable attention. The COD deficit is an effective indicator for assessing lower limb asymmetry [14,17]. It appears that team sports players who demonstrate greater limb symmetry (assessed via unilateral vertical jump or distance reached during a dynamic balance test) are faster than their asymmetrical counterparts during COD sprint tests [18]. Spiteri et al. reported that professional female basketball players demonstrate better lower limb balance than collegiate female basketball players [19]. Additionally, the injured side of recreational team sport players was found to be 2% slower than the healthy side in the 505 COD test [18]. Among basketball players, combined unilateral training effectively reduced lower limb asymmetry and enhanced COD capability [20]. It has also been observed that during double-leg landing tasks, volleyball athletes may be predisposed to unilaterally higher ground reaction or muscle forces, ultimately increasing the risk of injury during landing [21]. These findings underscore the importance of reducing lower limb imbalances to execute more proficient on-court movements.

Samozino et al. have recently developed a method to assess the entire force–velocity (Fv) spectrum during sprint acceleration (sprint Fv profile), profiling the mechanical capabilities of the neuromuscular system [22,23]. Since the sprint Fv relationship is linearity, the maximal capacities of muscles to produce force (F_0_), velocity (V_0_), power (P_max_), Fv_slope_ (the ratio between F_0_ and V_0_), maximal ratio of force (RF_max_), maximum speed during a sprint (V_max_) and the index of force application technique (D_RF_) can be determined within a linear regression model [23,24]. The sprint Fv profile has already been established as a reliable theoretical basis for devising personalized training guidance for athletes [22,25,26,27,28]. An athlete engaged in sports with lower speed demands may benefit from more maximal speed sprint training, while those exhibiting lower horizontal force outputs may require additional horizontal force training [29]. Additionally, studies have indicated that sprint Fv profile variables contribute independently to explaining COD performance in basketball and volleyball [30,31]. Thus far, only three studies have explored the relationship between COD ability and the Fv profile variables in volleyball and basketball. One study found that the F_0_, Fv_slope_ and RF_max_ had low to moderate correlations (0.32–0.54) with the COD deficit in volleyball players [30]. Another study revealed a moderate to strong negative correlation (r = −0.569 to −0.794) between the 505 COD test times of the dominant and non-dominant legs and the variables F_0_, V_0_, P_max_ and F_0_ within the Fv profile in 15 basketball players [31]. Furthermore, research on basketball players showed that F_0_, RF_max_ and P_max_ are the most determinant sprint Fv profile variables for greater COD performance and minimizing the COD deficit [32]. Studies on the association between COD performance and sprint Fv profile variables in basketball players have produced varying conclusions. In contrast, the limited number of studies on volleyball players has left the relationship between COD performance and sprint Fv profile variables unclear and inconclusive. Meanwhile, currently no research has examined the connection between asymmetries in COD speed performance and the sprint Fv profile in basketball and volleyball players. However, improving COD ability and reducing lower limb imbalance during the COD performance are crucial for volleyball and basketball players. The purpose of this study was to assess the associations between sprint Fv profile variables with COD performance and to investigate the impact of these variables on the asymmetries in COD speed performance.

## 2. Materials and Methods

### 2.1. Participants

Ninety-nine team sports collegiate players were selected, including forty-four volleyball players (age: 20.55 ± 1.88 years; height: 176.56 ± 5.21 cm; body mass: 82.55 ± 9.20 kg; BMI: 23.24 ± 2.55; training years: 7.64 ± 1.56) and fifty-five basketball players (age: 20.32 ± 2.41 years; height: 179.95 ± 8.41 cm; body mass: 75.89 ± 10.15 kg; BMI: 23.38 ± 2.10; training years: 8.48 ± 2.83). All the participants participated in an average of 10 h per week of combined team practice and technical skills, plus one competitive match per week. Additionally, they participated as key players in provincial-level or higher competitions in China, achieving top-three finishes. Exclusion criteria were musculoskeletal injuries within the last 6 months and traumatic surgeries within the last 12 months. None of the participants had any injuries or limitations that could affect their testing performance. Written informed consent was obtained from all subjects, and the study was approved by the ethics committee of Beijing Sport University (approval number 2023211H).

### 2.2. Study Design

A descriptive cross-sectional design was used to determine the relationships between the sprint Fv profile (F_0_, V_0_, P_max_, Fv_slope_, RF_max_, D_RF_ and V_max_) and COD performance. Before the testing, all participants were familiarized with the experimental procedures and completed two tests separated by at least 24 h and no more than 7 days. During the first test, participants performed the sprint Fv profile test. During test two, participants performed the COD performance test (the order of the COD performance tests was randomized, and sufficient rest was ensured between each test). All tests were performed indoors at a similar time of day to avoid effect of the circadian rhythm and under controlled conditions (i.e., temperature: min 20 °C, max 33 °C; atmospheric pressure: 1016 hPa). Participants were required not to engage in strenuous exercise within the 24-h period preceding the testing (i.e., no professional practice; only dynamic mobility was allowed). All participants completed three 40 m sprints, three MATs and six attempts of 505 COD tests with both the dominant and non-dominant legs. Before testing, all participants performed a standardized warm-up, starting with a 5-min jog, followed by 5 min of low-intensity sprints and ending with 5 min of dynamic stretching.

### 2.3. Sprint Force–Velocity Profile Test

A standardized warm-up was performed before the test. Six pairs of photocells (Smart Speed; Fusion Sport, Brisbane, Australia) were positioned at the starting line and at distances of 10, 20, 25, 30 and 40 m at approximately 1.2 m high, to measure the intervals of 0–10 m, 0–20 m, 0–25 m, 0–30 m and 0–40 m [24,33]. The sprint test was conducted on an indoor running track. Participants started from a standing position 0.5 m behind the starting gate and then sprinted at full effort through the finish line, performing the test three times with a 5 min rest interval between each, and the time was recorded to the nearest 0.001 s. The sprint Fv profile characteristics (F_0_, V_0_, P_max_, Fv_slope_, RF_max_, D_RF_, V_max_) were computed with the mean of three sprint split times according to Samozino’s specific spreadsheet [23,24]. The mean 0–10 m split time was used to calculated the COD deficit and asymmetry index, the process of which will be detailed later.

### 2.4. COD Performance Tests

#### 2.4.1. Modified Agility T-Test (MAT)

**Figure 1:**
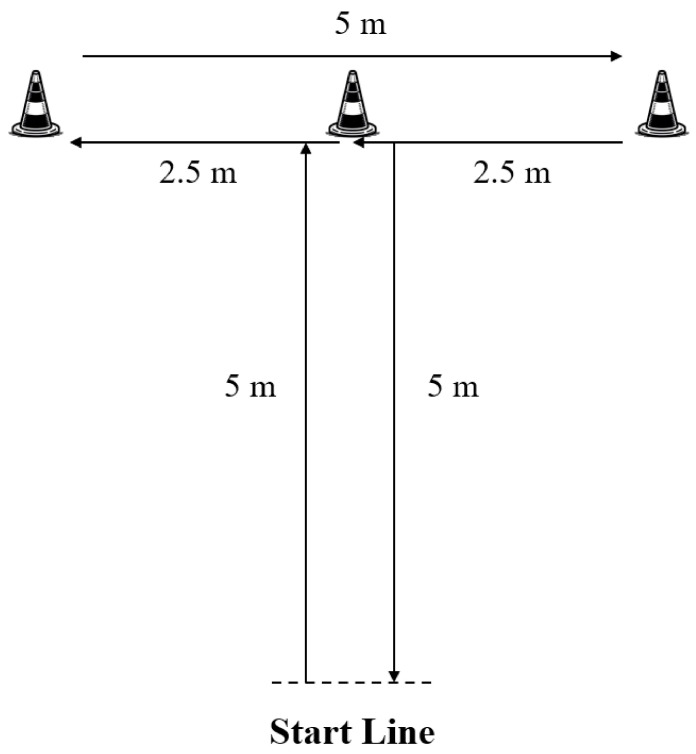
The structure of the Modified Agility T-test, m = meters.

A pair of photocells (Smart Speed; Fusion Sport, Brisbane, Australia) was positioned at the start line. Participants started from 0.5 m behind the starting gate and performed the MAT, which included liner sprinting and multidirectional running (Figure 1) [7]. Initially, participants completed a forward 5 m linear sprint to touch the landmark by hand, followed by a lateral, leftward shuffle for 2.5 m to touch the landmark, then a lateral, rightward shuffle for 5 m to touch the landmark, a lateral, leftward shuffle for 2.5 m to touch the middle landmark and, finally, a linear backpedal for 5 m. Each test was performed three times, and a 2 min rest interval was allowed between tests; the time was recorded to the nearest 0.001 s. The fastest times to complete the test were used for the subsequent analysis.

#### 2.4.2. 505 COD Test

**Figure 2:**
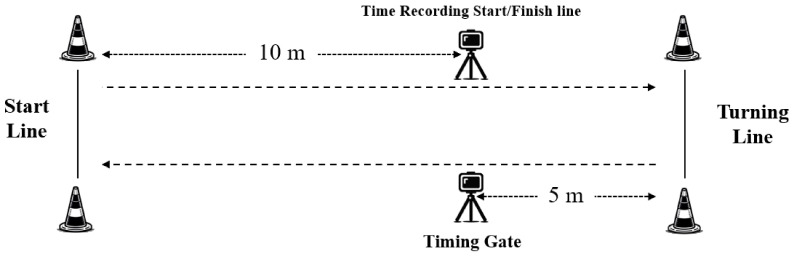
The structure of the 505 Change of Direction test, m = meters.

The participants were required to sprint with full effort to a line placed 15 m from the start line and performed a 180° turn, and then sprint 5 m through the finish line (Figure 2). Each participant completed six trials with 2 min of recovery between trials (three turning off the right leg and three off the left leg); the order of trials was randomized amongst the participants [16]. A pair of photocells (Smart Speed; Fusion Sport, Brisbane, Australia) was positioned at the 10 m mark, and time was recorded to the nearest 0.001 s. Participants started from 0.5 m behind the starting line, sprinted at full speed to the turning line, then performed a 180° turn with either the left or right leg randomly, and sprinted at full speed to the 5 m finish line to complete the trial. If a participant changed direction or turned off the incorrect foot before reaching the turning line, the result of that attempt would be disregarded. Participants were required to fully rest before beginning the test again [14]. The fastest times to complete the distance with each leg were calculated for the COD deficit and asymmetry index.

### 2.5. Data Processing

The COD deficit was calculated by subtracting the 10 m sprint from the 505 COD test time [14].The dominant (D) COD speed performance was defined as the leg side with the fastest completion time, while the non-dominant (ND) COD speed performance was the leg side with the slower completion time [14]. The COD deficit for dominant and non-dominant limbs was calculated using the following formula: 505 COD test time—10 m sprint time; the 10 m sprint time was taken from the 40 m split (the 0–10 m split time) [15,17,34]. Meanwhile, the asymmetry index was determined via the COD deficit of each leg. The COD deficit imbalance were asymmetry indices to evaluate lower limb balance during COD in participants. Calculated using the COD deficits of the D and ND limbs, the asymmetry index calculation formula is as follows: (D − ND)/D × 100 [14,34,35].

### 2.6. Statistical Analyses

Participants’ baseline data were reported as mean ± standard deviation (SD), and other continuous variables were presented as medians with interquartile range (IQR) depending on the distribution of the data. The Shapiro–Wilk test was used to assess the normality of the variables. Collinearity was evaluated for each variable using the variance inflation factor (VIF), and variables with VIF ≥ 10 were considered collinear [36].

Due to the non-normality and multicollinearity of the datasets, a partial least squares (PLS) regression analysis was conducted. This analysis included explanatory variables from the mechanical characteristics of the Fv sprint profile (F_0_, V_0_, P_max_, RF_max_, D_RF_, Fv_slope_ and V_max_) and gender. It also considered COD performance indicators such as COD deficit imbalance and the completion times of the MAT, 505-D, 505-ND, COD deficit D and COD deficit ND tests as different response variables. This approach aimed to explore the deeper relationship between the Fv sprint profile and COD performance. Composite variables constructed by PLS were used to build a linear regression model to identify which of these composite variables, encompassing different potential variables, significantly predict COD performance, with a *p*-value < 0.050 considered significant for composite variables [37].

Different composite variables significantly predicted various response variables for COD performance. The PLS regression coefficients (β) for the original explanatory variables within these composite variables were then estimated, along with standard error (SE) and bias-corrected and accelerated (bca) 95% confidence intervals (Cls) after bootstrapping. The SE and 95%CIs for coefficients in the PLS regression were estimated using the bootstrap method, an agnostic estimation method that does not rely on model form assumptions [38,39,40]. The number of bootstrap iterations was set to 2000 [41,42]. Explanatory variables were considered significant if zero was not included in the 95% bcaCIs [43,44]. The larger the absolute value of the coefficient of an explanatory variable, the greater the influence on the response variable relative to other Fv profile explanatory variables in the composite variable [45]. The optimal number of components in the PLS regression was determined by comparing the cross-validation root mean square error of prediction (RMSEP) across different numbers of components [45]. Statistical analyses were performed using the R (version 4.3.2). The following packages were utilized:pls, ggplot2, showtext, gridExtra.

## 3. Results

Table 1 shows the variables within the sprint Fv and COD performance tests.

**Figure 3:**
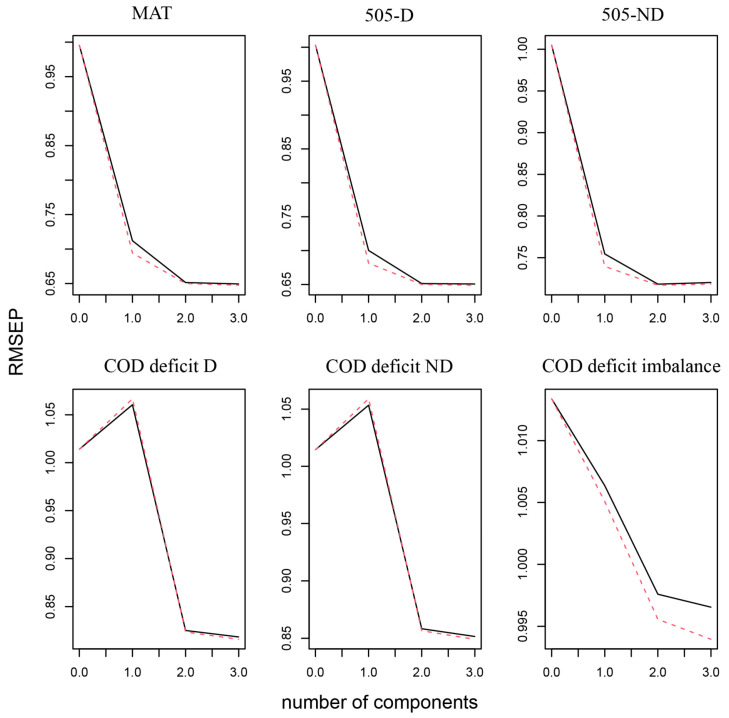
Root mean square error of prediction (RMSEP) for different numbers of components of partial least squares (PLS) regression.

The RMSEP was plotted against the number of components used in the PLS regression analysis in Figure 3, which suggested that two components should be included in the PLS regression model (Figure 3); meanwhile, two components were chosen because adding a third component did not lead to an increased adjusted R^2^. Component 1 comprised a linear combination of gender, V_0_, P_max_, RF_max_, D_RF_, Fv_slope_ and V_max_, while component 2 was formed from a linear combination of F_0_, P_max_, RF_max_, D_RF_ and Fv_slope_.

Table 2 presents results from a linear model predicting COD performance using two PLS components. The results revealed that the effect of component 1 was significantly negatively associated with the performance of MAT, 505-D and 505-ND (*p* < 0.05). The effect of component 2 was significantly positively associated with COD deficit D and COD deficit ND (*p* < 0.05). Additionally, the effects of components 1 and 2 were both significantly correlated with COD deficit imbalance (*p* < 0.05).

**Figure 4:**
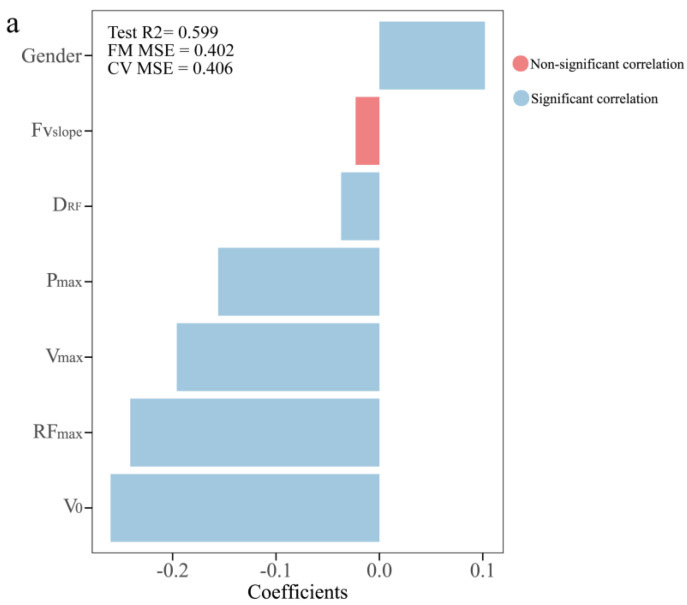

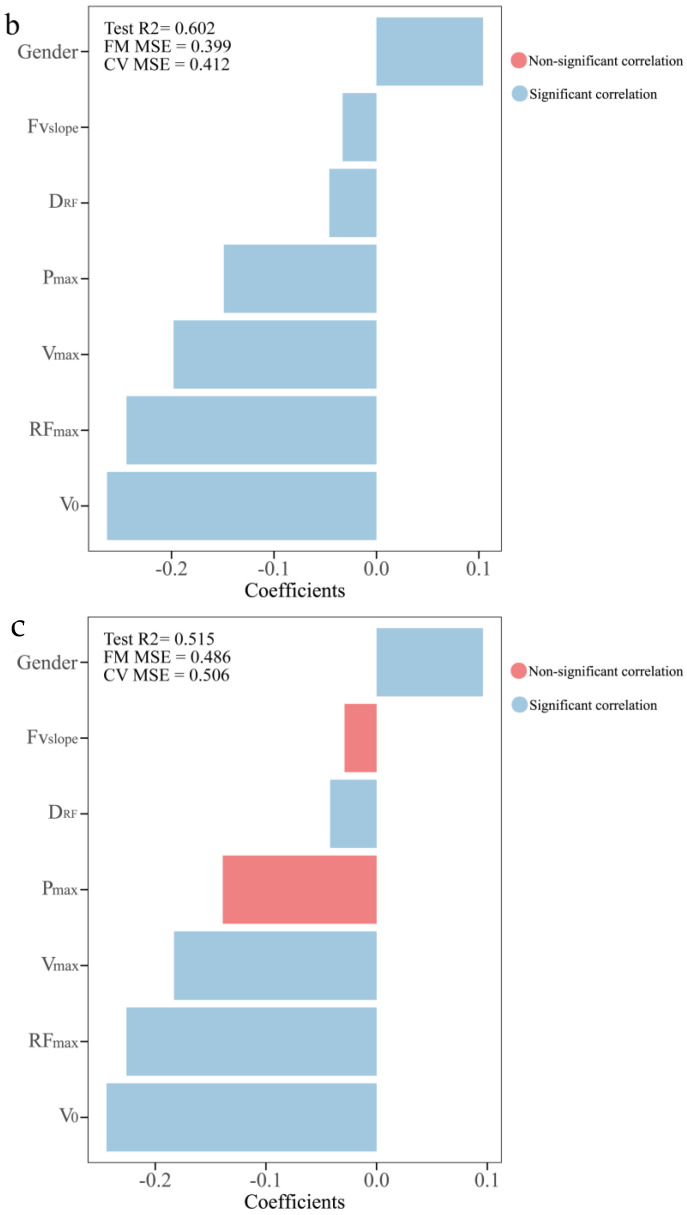
PLS regression coefficients between sprint Fv profile variables and COD performance. (**a**) MAT time, (**b**) 505−D time, (**c**) 505−ND time. R^2^: coefficient of determination; FM MSE: folded mean square error; CV MSE: cross-validated mean square error.

**Figure 5:**
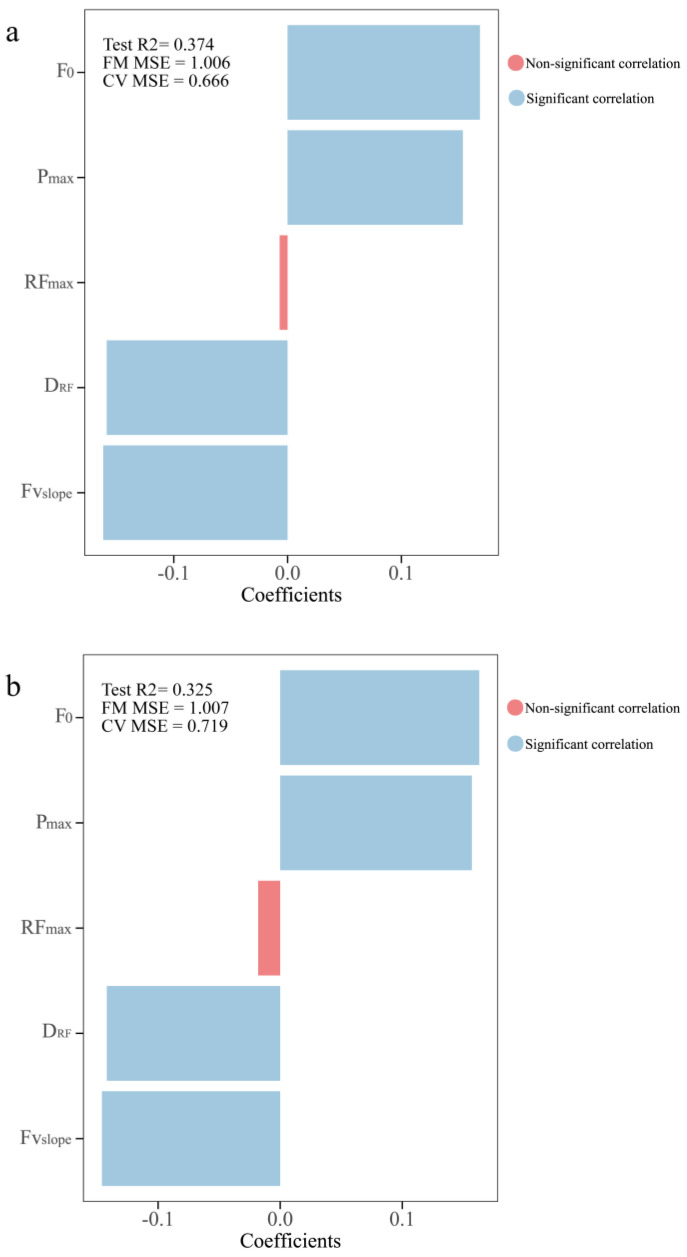

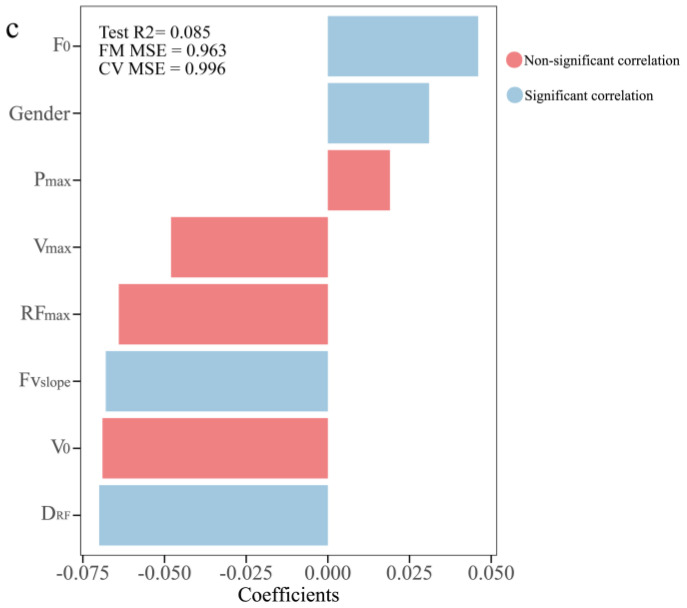
PLS regression coefficients between sprint Fv profile variables and COD performance. (**a**) COD deficit D time; (**b**) COD deficit ND time; (**c**) COD deficit imbalance time. R^2^: coefficient of determination; FM MSE: folded mean square error; CV MSE: cross-validated mean square error.

Figure 4 and Figure 5 present the relationship between the sprint Fv profile variables and COD performance in PLS regression. Figure 4 displays the correlations between the response variables significantly related to component 1 and the explanatory variables that composed component 1 in the PLS model. Gender (95% bcaCl = [0.08 to 0.13], [0.08 to 0.13], [0.07 to 0.12]) was significantly correlated with COD performance variables (MAT, 505-D and 505-ND). In contrast, V_0_ (95% bcaCl = [−0.30 to −0.21], [ −0.30 to −0.21], [−0.29 to −0.19]), RF_max_ (95% bcaCl = [−0.28 to −0.20], [−0.29 to −0.20], [−0.27 to −0.17]), V_max_ (95% bcaCl = [−0.23 to −0.16], [−0.23 to −0.16], [−0.22 to −0.15]) and D_RF_ (95% bcaCl = [−0.07 to −0.01], [−0.08 to −0.02], [−0.08 to −0.02]) showed significant negative correlations with COD performance variables (MAT, 505-D and 505-ND). The V_0_ was the most influential variable negatively associated with COD performance variables (β = −0.260, −0.263 and −0.244 for MAT, 505-D and 505-ND, respectively), followed by RF_max_, V_max_, gender and D_RF_.

Figure 5a,b show the correlations between the response variables significantly related to component 2 and the explanatory variables that comprised component 2 in the PLS model. Except for RF_max_, all the variables had significant associations with COD performance. F_0_ (95% bcaCl = [0.13 to 0.22], [0.12 to 0.22]) and P_max_ (95% bcaCl = [0.11 to 0.21], [0.11 to 0.22]) were significantly positively associated with COD performance variables (COD deficit D and COD deficit ND), while D_RF_ (95% bcaCl = [−0.21 to −0.12], [−0.19 to −0.10]) and Fv_slope_ (95% bcaCl = [−0.21 to −0.13], [−0.20 to −0.11]) were negatively significant associated with COD performance variables (COD deficit D and COD deficit ND). The effect of F_0_ (β = 0.169, 0.163) on the COD performance variables (COD deficit D and COD deficit ND) was slightly larger than the effects of Fv_slope_ (β = −0.162, −0.146), D_RF_ (β = −0.159, −0.142) and P_max_ (β = −0.162, −0.146).

Figure 5c displays the correlations between the response variables significantly related to two components and the explanatory variables that comprised these two components in the PLS model. Gender (95% bcaCl = [0.00 to 0.60]) and F_0_ (95% bcaCl = [0.00 to 0.10]) were significantly positively associated with COD deficit imbalance, while D_RF_ (95% bcaCl = [−0.12 to −0.03]) and Fv_slope_ (95% bcaCl = [−0.11 to −0.02]) were significantly negatively associated with COD deficit imbalance. The D_RF_ (β = −0.070) was the most influential variable followed by Fv_slope_ (β = −0.068), F_0_ (β = 0.046) and gender (β = 0.031).

## 4. Discussion

This aim of this study was to evaluate associations between sprint force–velocity profile variables and COD performance and to investigate the impact of these variables on the asymmetries in COD speed performance for basketball and volleyball players using PLS regression analysis. The main findings indicated that, within the Fv profile variables, V_0_ and RF_max_ were significant predictors affecting performance in COD movement patterns that included linear sprints, while F_0_, P_max_, D_RF_ and Fv_slope_ were significant predictors impacting COD deficit. D_RF_ and Fv_slope_ were significant predictors for assessing asymmetry in COD speed performance using the COD deficit time. Sprint Fv profile variables appeared to be helpful in explaining COD capability.

The MAT and 505 COD test involved athletes performing a linear sprint before initiating a COD movement at angles of 90° and 180°, followed by a subsequent linear sprint [7,14]. The COD deficit was calculated by subtracting the time for a 10 m sprint from the total 505 COD test time, which independently evaluates the performance of the COD movement [16]. This might also have been the reason why the predictive variables for COD deficit and 505 COD test time differ in this study. Studies have shown that the speeds of 10 m and 30 m linear sprints significantly impacted the total time of the 505 COD test. Meanwhile, in the MAT, both COD speed and linear sprinting jointly determine the final MAT time [16,46]. Linear sprinting involves the movement of the body’s center of mass (CoM); the forward acceleration of the CoM from one step to another is directly related to the net force developed by the athlete onto the ground in the horizontal, anteroposterior direction. Research has shown that greater agility and T-test athletes demonstrated significantly greater propulsive impulse compared with slower athletes. Faster athletes during the modified 505 test produced greater horizontal propulsive force in shorter ground contact times, and under constant conditions, the greater the net horizontal force relative to body weight, the higher the forward acceleration of the body, and the anteroposterior force was identified as a critical factor in enhancing sprint acceleration and related performance metrics [47,48,49]. Studies indicated that while the acceleration phase was highly related to V_0_, as well as to averaged velocity and power measured in the forward direction obtained in the Fv relationship, F_0_ was not significantly correlated with performance parameters during the acceleration phase. Additionally, the Fv relationship showed that elite sprinters are able to produce higher horizontal force than sub-elite sprinters at any velocity [50]. Therefore, RF_max_ and V_0_ were particularly important factors affecting the performance of COD movement patterns that included linear sprints.

In badminton, boys performed better than girls in the modified 505 test [51]. And in rugby, male athletes significantly outperformed female athletes in all COD tests (5-10-5, L-drill) [52]. Similar to these findings, we found that the times for the MAT as well as the 505 COD tests for dominant and non-dominant legs showed a significant positive correlation with gender, with males having shorter times than females; this may be related to differences between males and females in muscle cross-sectional area and the capacity to recruit muscle fibers [53,54]. Another study examining the impact of gender on the correlation between sprint Fv variables and COD capability in basketball players indicated that F_0_, RF_max_ and Pmax affected the 505-D and 505-ND performance of both male and female basketball players, and V_0_ was also significantly correlated with 505-D and 505-ND performance in female basketball players [32]. In a study examining the relationship between the Fv profile and linear sprint and COD performance across multiple sports (soccer, tennis and basketball), the 505-D and 505-ND times were significantly negatively correlated with sprint Fv-related variables F_0_, V_0_ and P_max_ [31]. These results were generally consistent with the findings of the present experiment, except for the correlation with the variable F_0_, which may be due to differences in the team sports, as the sports in this study focused on team sports with intensive directional changes on smaller fields.

When independently evaluating the 180° COD ability using the COD deficit metric, it was found that F_0_ and P_max_ were negatively correlated with COD capability, while D_RF_ and Fv_slope_ were positively correlated. When changing direction, athletes had to rapidly apply force during the braking phase (eccentric), plant phase (isometric) and propulsive phase (concentric) of movement [47]. Braking and propulsive forces are crucial factors affecting the performance of 180° COD movements. In the COD 505 test, faster athletes produced significantly greater braking and propulsive force compared with slower athletes [47]. Increasing force application during the braking and propulsive phases of COD movements has been shown to increase exit and starting velocities during the COD movement because muscles initially underwent eccentric contractions and were passively elongated to do negative work, which increased the storage of mechanical energy in their elastic components. Subsequently, they performed concentric contractions, enabling the muscles to provide significant braking and propulsive force during COD movements [49]. Moreover, studies have shown that compared to 90° changes of direction, 180° changes require more braking and propulsive time [47]. Consequently, in 180° COD movements, athletes need to maintain sufficient braking and propulsive force over a longer contact time (braking time and propulsive time) to ensure a smooth 180° COD. Thus, a smaller absolute value of D_RF_ can reduce COD deficit, improving performance in 180° COD movements. In basketball players, D_RF_ was significantly negatively correlated with the COD deficit in female basketball players and the dominant leg COD deficit in male basketball players, which is generally consistent with the findings of this study [32].

Research has found that faster youth netball athletes had longer COD deficit times and may not have had the capability to efficiently decelerate, change direction by 180° and reaccelerate, and that eccentric strength was essential for COD ability, especially during the braking phase [15,55]. While F_0_ may optimize performance in linear sprints, it is not entirely applicable to COD movements. Additionally, a higher F_0_ might lead to propulsive force exceeding braking force, thus requiring athletes to recruit more neuromuscular fibers during the braking phase to generate greater eccentric force for deceleration, which, in turn, increases COD deficit time. Furthermore, an increase in F_0_ also increases P_max_; hence, higher values of both F_0_ and P_max_ were associated with a higher COD deficit. One study indicated that a higher F_0_ was associated with a lower COD deficit, while another study showed that a higher F_0_ was associated with a higher COD deficit on the right leg [30,32]. This discrepancy could be due to differences in the COD techniques of the participants in the experiments. Training should focus on both strength and skill enhancement to increase the utilization rate of F_0_ during a 180° COD. The Fv_slope_ indicates an athlete’s acceleration performance, with a theoretical optimal slope that can maximize acceleration performance and thus minimize COD time. An Fv_slope_ that is too high compared to the optimal slope can reduce the ability to maintain horizontal force, while too low a slope can decrease the average horizontal output power, both affecting the athlete’s performance [33]. In this study, a smaller absolute value of Fv_slope_ was associated with a better COD deficit. This result aligns with the significant correlation found between higher F_0_ and poorer COD deficit, as a higher F_0_ corresponds to a larger absolute value of Fv_slope_. One possible explanation is that, although a high F_0_ increases output force, it lacks the capacity to maintain force under high-intensity output.

A study examining the association between Fv profile variables and specific performance metrics in volleyball players indicated that F_0_, Fv_slope_ and RF_max_ were significantly correlated with COD deficit performance. COD right deficit was significantly correlated with D_RF_, and P_max_ obtained during sprints was closely related to 505 left performance, but not significantly related to 505 right [30]. This was generally consistent with our results; however, in our study, the predictors for dominant and non-dominant legs under COD deficit assessment showed better uniformity. Similarly, another study found that female collegiate soccer athletes performing a COD (cutting) task with their dominant and non-dominant legs exhibited similar movement patterns [55]. Meanwhile, our findings revealed the predictors for the dominant and non-dominant sides showed differences in P_max_ during the total 505 COD time evaluation, reflecting the variations in the ability of the two legs to generate horizontal force across the entire speed range. This discrepancy may have been caused by uneven force production by the legs during linear sprints.

COD deficit asymmetries, which independently evaluate asymmetries in COD speed performance, could be interpreted as a deficiency in COD ability on one side, indicating that an athlete has a faster or slower side when performing a 180° COD [15]. This is disadvantageous for multidirectional sports, as proficiently changing direction from both limb directions equally could enhance performance in gameplay like basketball and volleyball. Our findings revealed that COD deficit asymmetries were significantly negatively correlated with D_RF_ and Fv_slope_. However, the effect size of the PLS regression was insufficient. Currently, only one study has investigated the relationship between lower limb asymmetry in jumping and the force–velocity profile. The study indicated that there was no or a very low linear relationship between the isokinetic knee force–velocity profile and unilateral jumps in basketball players [56]. Assessments should consider specific COD in gameplay, including angles and whether they include certain short-distance linear sprints, to determine the asymmetry in COD speed performance and influencing factors [2]. Currently, no studies have reported on the relationships between asymmetries in COD speed performance and Fv profile variables. A study on lower limb injuries has reported that gender plays a significant role in knee joint mechanics during COD tasks (cutting and pivoting) and was one of the risk factors for knee injuries [57]. Consistent with the results of this study, gender was a significant predictor affecting COD deficit imbalance.

This study has limitations that must be highlighted. Firstly, it is a cross-sectional study, and these results require further comparison in future prospective studies to assess whether COD performance can be enhanced by optimizing Fv profile-related variables. Secondly, COD movements on the sports field are complex processes. Our finding has already explored the association between maximum force and maximum horizontal force produced during sprints and COD performance. Future research can combine biomechanical analysis to explore the relationships between mechanical characteristics, such as the penultimate and final foot contacts in COD performance, and the sprint Fv profile variables. Lastly, we conducted a preliminary analysis of the relationships between asymmetries in COD speed performance and sprint Fv profile variables. Future research can refine the analysis of Fv profile variables and asymmetries by incorporating biomechanical analysis.

## 5. Conclusions

Investigating the relationships between COD ability and sprint Fv profile variables in team sports played on smaller courts, such as basketball and volleyball, can provide new insights for personalized training based on sprint mechanical characteristics to enhance COD performance. According to our findings, RF_max_ and V_0_ among the sprint Fv profile variables are significantly associated with COD performance that includes linear sprints, while D_RF_, Fv_slope_, F_0_ and P_max_ collectively affect the 180° COD performance. Concurrently, the D_RF_ and Fv_slope_ within sprint Fv profile variables can partly explain asymmetries in COD speed performance. In the future, training interventions could be tailored by identifying the specific COD performance patterns, including the angle of change and the distance of the linear sprint, by determining the related sprint Fv profile variables, thereby providing guidance for enhancing specific COD performance.

## 6. Practical Application

Current research evidence suggests that developing personalized training based on force–velocity (Fv) profile variables to improve change of direction (COD) performance in basketball and volleyball players may be an effective option for coaches and researchers. This study also innovatively explores the correlation between sprint lower limb asymmetry and the sprint force–velocity profile in basketball and volleyball athletes. Among the assessed subjects, asymmetries in COD performance were indeed present. The results of this study provide preliminary theoretical evidence supporting the use of Fv profile-based personalized training by coaches to reduce asymmetries in COD performance.

## Figures and Tables

**Table 1:** Descriptive statistics for COD performance variables and Fv sprint profile variables (n = 99).

Variables	Medians (IQR)
Sprint Fv profile	
F_0_ (N·kg^−1^)	12.96 (8.33, 28.12)
V_0_ (m·s^−1^)	7.97 (5.47, 9.33)
P_max_ (W·kg^−1^)	26.43 (12.17, 56.10)
RF_max_	0.50 (0.37, 0.55)
D_RF_	−0.15 (−0.35, −0.10)
Fv_slope_ (N·s·m^−1^kg^−1^)	−1.64 (−3.61, −1.09)
V_max_ (m/s^−1^)	7.84 (5.41, 9.02)
COD Performance tests	
Time to 10 m (s)	1.82 (1.50, 2.41)
MAT (s)	5.73 (4.84, 7.67)
505-D (s)	2.36 (1.99, 2.94)
505-ND (s)	2.43 (2.11, 3.02)
COD deficit D (s)	0.53 (0.23, 1.07)
COD deficit ND (s)	0.61 (0.24, 1.08)
505 COD imbalance (%)	−2.58 (−7.4, −0.04)
COD deficit imbalance (%)	−11.22 (−34.89, −0.12)

IQR: interquartile range; F_0_: theoretical maximal force production; V_0_: theoretical maximal running velocity; P_max_: theoretical maximal mechanical power in the horizontal direction; Fv_slope:_ force–velocity slope; RF_max_: maximum ratio value of horizontal component to resultant force; D_RF_: index of force application technique; V_max_: maximal velocity; COD: change of direction; D: dominant; ND: non-dominant; m: meters.

**Table 2:** Coefficients from linear regression model of partial least squares (PLS) composite variables predicting COD performance.

		β (SE)	T	*p*-Value	adj-R^2^
MAT	Intercept	−0.034 (0.06)	−0.535	0.594	0.59
	Comp1	−0.451 (0.04)	−11.94	<0.001 *	
	Comp2	−0.028 (0.03)	−0.842	0.402	
505-D	Intercept	−0.019 (0.06)	−0.294	0.770	0.59
	Comp1	−0.456 (0.04)	−12.053	<0.001 *	
	Comp2	−0.010 (0.03)	−0.303	0.763	
505-ND	Intercept	−0.017 (0.07)	−0.239	0.812	0.50
	Comp1	−0.423 (0.04)	−10.083	<0.001 *	
	Comp2	−0.011 (0.04)	−0.305	0.761	
COD deficit D	Intercept	−0.005 (0.08)	−0.057	0.954	0.36
	Comp1	0.004 (0.05)	0.089	0.929	
	Comp2	0.322 (0.04)	7.571	<0.001 *	
COD deficit ND	Intercept	−0.002 (0.08)	−0.025	0.980	0.31
	Comp1	0.050 (0.05)	1.01	0.315	
	Comp2	0.297 (0.04)	6.727	<0.001 *	
505 COD imbalance	Intercept	−0.008 (0.10)	−0.081	0.935	0.05
	Comp1	−0.151 (0.06)	−2.582	0.011 *	
	Comp2	−0.005 (0.05)	−0.098	0.922	
COD deficit imbalance	Intercept	−0.009 (0.10)	−0.088	0.930	0.07
	Comp1	−0.122 (0.06)	−2.115	0.037 *	
	Comp2	0.109 (0.05)	2.103	0.038 *	

Comp: component; β: standardized regression coefficient; SE: standard error; adj-R^2^: adjusted coefficient of determination; COD: change of direction; D: dominant; ND: non-dominant. * Significant correlation (0 was not included in the 95% bcaCI).

## Data Availability

The data of this study are available from the author upon request.
